# ‘No One Has Taught Us to Have It All’: Reflections from Women on the Gender-Based Challenges in Surgical Careers in Africa

**DOI:** 10.24248/eahrj.v8i3.815

**Published:** 2025-01-30

**Authors:** Marcella F.D. Ryan-Coker, Mwanaidi Ayumba, Lotta Velin, Ala Magzoub, Joselyne Nzisabira, Grace Paidamoyo Gwini, Samuel Mesfin Girma, Aemon Berhane Fissha, Amy Paterson

**Affiliations:** a Department of Surgery, University of Nairobi, Nairobi, Kenya; b Deanery of Clinical Sciences, University of Edinburgh, Edinburgh, Scotland; c Department of Human Anatomy and Physiology, Pwani University, Kenya; d Centre for Teaching & Research in Disaster Medicine and Traumatology (KMC), Department of Biomedical and Clinical Sciences, Linköping University, Linköping, Sweden; e Faculty of Medicine, University of Gezira, Sudan; f School of Medicine, University of Global Health Equity, Rwanda; g School of Public Health, University of Cape Town, South Africa; h College of Health Sciences, Addis Ababa University, Addis Ababa, Ethiopia; i Division of Global Surgery, University of Cape Town, South Africa; j Pandemic Sciences Institute, University of Oxford, Oxford, United Kingdom.

## Abstract

**Background:**

The surgical field in Africa has long grappled with a gender imbalance, with women being significantly underrepresented. Despite global efforts to foster gender diversity in healthcare practices, African women pursuing surgical careers still face substantial hurdles. This paper investigates these women’s experiences and challenges, aiming to raise awareness of these issues and propose strategies for improving gender equity.

**Objective:**

To describe contextual aspects of barriers affecting women in surgery in Africa.

**Methods:**

A cross-sectional survey was conducted, targeting female medical students interested in surgical careers, interns, trainees, and surgical consultants across Africa. The survey was distributed in November-December 2021. Data were analysed using descriptive statistics for quantitative data and a simplified thematic analysis for qualitative data.

**Results:**

A total of 105 participants from 17 countries, aged 20 to 50 years and with various training levels, completed the survey. General surgery was the most common speciality among the respondents. Notably, 63% reported gender-based discrimination, with many (74%) attributing societal and familial discouragement and financial commitments as major barriers to pursuing surgical careers. Participants also shared experiences of gender-based inequity, underestimation of their skills, sexist comments, and even instances of sexual harassment during training or work.

**Conclusion:**

This study sheds light on the complex barriers African women face in pursuit of surgical careers. To enhance diversity in the field, fundamental change is required. This necessitates recognising the underlying causes hindering women’s progress in surgery and the implementation of interventions to promote gender equity.

## BACKGROUND

In medicine, the surgical profession symbolises precision, courage, and life-saving innovations. However, for women in surgery, the path to becoming a surgeon remains strewn with numerous and often profoundly ingrained obstacles. Throughout history, the surgical arena globally has been predominantly the domain of men.^1^ Surgical training demands an unwavering commitment to rigorous training and access to opportunities, hence why it is often argued to be a better career fit for men.^2,3^

Africa’s unique cultural tapestry, socioeconomic disparities, and evolving healthcare landscapes have given rise to a distinct set of hurdles for women with surgical aspirations. The continent faces a severe shortage of specialist surgical workforce, owning only ~12% of the global surgical workforce despite hosting over a third of the population.^4,5^ Although women in surgery in Africa have come a long way from being about 9% to about 23% of all practicing surgeons on the continent, there is still a significant gap.^4,6^ Societal norms, traditional gender roles, limited access to quality education, workplace bias, and a dearth of female surgical mentors collectively contribute to this gender gap.^7,8^ Most existing studies on this issue in the region are focused on individual countries. This multinational survey aims to describe contextual aspects of barriers and obstacles affecting women in surgery in Africa.

## METHODS

This cross-sectional multinational online survey was conducted as part of the work of the Gender Equity Initiative in Global Surgery (GEIGS). We surveyed female medical students interested in surgical specialities, interns, medical officers, registrars, and consultants in various surgical fields across Africa.

Ethical approval was obtained from the Sierra Leone Ethics and Scientific Review Committee. Informed consent was obtained from all participants before they engaged with the online questionnaire.

A 26-item questionnaire, set up on Google Form surveys and administered in three languages (English, French, and Arabic), was used to collect data from November to December 2021 via various online platforms. The questionnaire encompassed single- and multiple-choice questions, Likert scale items, and open-ended free-text responses. The survey collected quantitative and qualitative data, including demographic data, surgical training experience, mentorship, and experiences of bias and barriers to surgical training.

Data were de-identified for confidentiality. Quantitative data were analysed using the Statistical Package for the Social Sciences (SPSS). Braun and Clarke’s simplified version of thematic analysis was used for qualitative data.^9^ Responses were organised into condensed “meaning units.” These meaning units were then grouped into sub-themes, which were further categorised into overarching themes. Direct quotations from the participants were incorporated to exemplify sub-themes and illustrate the voice of the participants.

## RESULTS

**Participant Demographics:** A summary of participant demographics is presented in Table 1. The study featured 105 respondents, representing a diverse cross-section of individuals, predominantly young (n=74, 70% under 30 years) and unmarried (n=78, 74%). A notable 21% (n = 22) with Christianity being the predominant religious affiliation (n = 73, 70%).

**Table 1:** Participants Demographic Characteristics

Demographic Data	Frequency	Percentage (%)
Age range (years)		
<30	74	70.5
30-40	27	25.7
40-50	4	3.8
Country of origin		
Kenya	23	22.1
Zimbabwe	18	17.3
Sudan	13	12.5
South Africa	11	10.6
Sierra Leone	10	9.6
Others	8	7.7
Current country of residency		
Kenya	25	24.0
Zimbabwe	17	16.3
Sudan	14	13.5
Sierra Leone	9	8.7
Others	8	7.7
Marital status		
Single	78	74.3
Married	22	20.9
Engaged	3	2.9
Separated	2	1.9
Religion		
Christian	73	69.5
Muslim	24	22.9
Not religious	4	3.8
Prefer not to say	4	3.8
Children		
No	83	79.1
Yes	22	20.9
Number of children		
1	12	54.5
2	6	27.3
3 or more	4	18.2

**Work characteristics of participants:** More than half of the respondents (n=56, 53%) were medical students interested in surgical careers, most of whom were in their 4th-6th year of study. The majority of respondents (79%) worked in public healthcare institutions, though some had roles in private practice. The common specialities were general surgery (n=20) and obstetrics and gynaecology (n=8). Regarding work hours, a substantial group (48.1%) worked 41–60 hours weekly (Table 2).

**Table 2:** Work Characteristics of Participants

Variable	Frequency	Percentage (%)
Training level		
Medical student	56	53.3
Registrar	10	9.5
House Officer/Intern	10	9.5
Medical officer	10	9.5
Specialist/Consultant	13	12.4
General practitioner	1	0.9
Independent practitioner	1	0.9
Not stated	3	2.8
Type of work institution*		
Public	38	79.2
Private	10	20.8
Mission/NGO	3	6.2
Surgical specialty		
General Surgery	20	41.7
Obstetrics & Gynaecology	8	16.7
Orthopaedics	5	10.4
Paediatrics	5	10.4
Neurosurgery	4	8.3
Plastic & Reconstructive Surgery	2	4.2
Urology	2	4.2
Cardiothoracic & Vascular Surgery	1	2.1
Surgical Oncology	1	2.1

* Some practitioners worked in more than one type of institution

**Gender Bias:** Sixty percent (n=63) of respondents acknowledge some form of gender bias in their training or work. Of these, 79% (n=50) reported the forms of biases experienced, which included competency underestimation (n=12, 24%), reduced opportunities for skill development and career progression (n=9, 18%), unsolicited advice (n=6, 12%), sexual harassment (n=3, 6%), and sexist jokes (n=2, 4%).

**Mentorship:** Mentorship was a critical factor in the experiences of the participants (n=96, 91%). However, only 36 (34%) respondents reported having a mentor, with a smaller fraction (n=17, 47%) having female mentors. Some of the perceived benefits of mentoring such as access to teaching resources and opportunities, assistance in career and personal life decision-making, increased confidence, and having a role model to aspire to.

### Thematic Analysis

**Figure 1:**
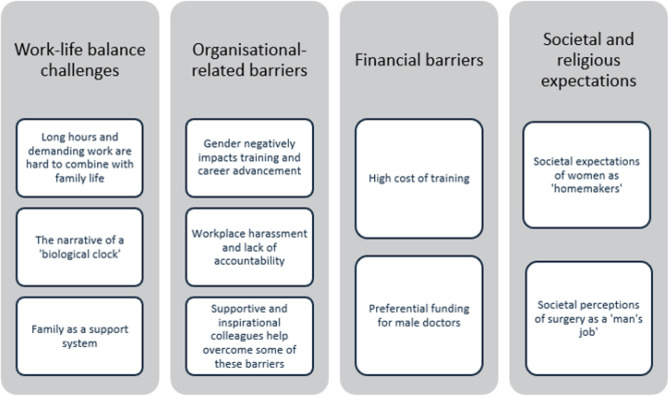
Analytical Model for Thematic Analysis

The inductive thematic analysis of the responses revealed four main themes (Figure 1).

#### Work-life balance challenges

This theme reinforces the delicate balance women in surgery must strike between demanding professional responsibilities and familial obligations. Respondents shared their experiences managing the extensive time and energy requirements of surgical training alongside the roles of mother and wife. A participant poignantly articulated, “Having to attend to emergencies at night, at the same time coming back home to the role of wife and mother, makes it difficult for spouses to accept that your job is demanding.”

Participants also reflected upon the tension of racing against the ‘biological clock,’ with societal pressures dictating timelines for marriage and family, often at odds with career aspirations. One respondent reported, “I have had to focus on work and training, so I am single. I have opted out of having children also, which is obviously frowned upon as an African.” Another said, “Society expects a ‘whole woman’ to be married and have kids at a certain age, like there are timelines to what a woman should be doing and when, hence the battle to become a societal woman and a professional woman at the same time.”

While societal expectations presented challenges, family and friends were perceived as potential sources of support. Participants acknowledged the critical role of their support systems in their surgical training, countering biases and challenges.

#### Organisational-related barriers

The theme of organisational-related barriers delved into experiences within medical schools, hospitals, or organisational settings. Respondents reported how gender influenced their training experience and professional progression, with women often viewed as inferior to their male counterparts. Comments such as: “Many women are looked down on or not given chances simply because of their gender” and “Being female, you are not always taken seriously. Surgery being considered a speciality for men and women not fully given a chance” illustrates some of the respondents’ lived experiences. Biases in training opportunities and clinical competency assessments emerged as key concerns, with reports such as: “Male counterparts are asked for advice first or given surgical opportunities before their female counterparts” or “at the hospital, women’s opinion is usually regarded second to that of men” or “Another assumption is that surgery requires physical strength, which females are incapable of,” highlighting the need for more inclusive practices. Descriptions of the work environment generally described the hostility faced by the respondents with comments such as: “The paramedic scrub nurse and male co-worker act with aggression towards the female surgeon or trainee.”

Workplace harassment, including sexual harassment, was a distressing reality faced by respondents at all levels of training, where the aggressors were typically at more senior levels. A medical student said, “…it is also disheartening to see incoming younger students being preyed on by men who are meant to be mentors and teachers.” The importance of reporting systems and accountability for perpetrators was emphasised. “Setting up a system in which people are held accountable for their actions. A system in which we can report or raise awareness of any form of harassment, with actual action being taken against perpetrators.”

Another sub-theme in the organisational barriers was the limited support for maternity leave and breastfeeding facilities.

Amidst the challenges, the theme of supportive colleagues in institutional settings shone through. The role of colleagues, particularly male colleagues, in providing vital support and mentorship was recognised as a powerful enabler. More senior women in leadership positions were seen as inspirational figures, driving change and motivation. One respondent remarked, “Seeing women of colour in positions of power, making big changes, is, and will always be, a massive driving push for me—if they could do it, I can too. I also often remind myself that it was probably much more difficult for them to achieve what they have given the times, and that they have lightened the load so that I may thrive too. This genuinely drives me.”

#### Financial barriers

The barrier of financial constraints was highlighted, including the high cost of surgical training and the limited financial resources. One respondent said, “The cost of training is ridiculous, and if no scholarships were available, I probably would not have come this far.”

Gender bias in funding allocation, favouring males, added another layer of complexity to the financial challenges faced by aspiring female surgeons. When financial support is limited, funding may preferentially be allocated to males in different levels of training. This was described both in early educational stages, which may reduce the likelihood of pursuing medical studies, and at more senior training levels. Respondents said, “Finances are directed more to the male child[ren]…” and “Financial barriers are one of the greatest challenges. Everybody, including my family, has distanced themselves. Because one medicine is expensive, and two, only men are expected to pursue sciences”. Another reported, “More often than not, young female doctors miss out on opportunities due to funds and societal pressure to settle down and take care of family. An easier path remains to give up or postpone the dreams.”

#### Societal and religious expectations

Societal and religious expectations were pervasive in participants’ lives, influencing personal and professional choices. Participants described the weight of societal expectations, where women were predominantly viewed as “homemakers,” impacting their ability to pursue surgical careers: “Women are viewed as homemakers more than career women, and social and family attachments make us lean more towards being homemakers; no one has taught us to have it all.” Another described these expectations: “Women are seen as helpers to their husbands, so they are expected by society to be home most of the time, taking care of their husbands and families… Men are the head, and women have to follow suit; therefore, they take on roles that are not demanding or require a high level of training.”

Furthermore, the perception of surgery as a “man’s job,” which was not compatible with the role of a wife and/or mother, added to the challenges: “Women in the medical profession feel like a surgical career is a demanding profession, and thus they will not have time to take care of/raise their families”. “People think women are not mentally or emotionally capable of practicing surgery, and they believe a woman should have to give up surgery to look after her family because a woman will not be able to handle both, so she has to sacrifice one, and that is simply not true, and if you end up going further in surgery, you are immediately described as a bad mother or wife or daughter or sister.”

The societal impact rippled through patients’ interactions, with respondents often mistaken for nurses while male nurses will be referred to as doctors due to entrenched biases. One respondent said, “Society never expects a female to be a doctor, let alone a surgeon.” Another respondent similarly highlighted the perceived belief that “patients will always prefer male surgeons.”

## DISCUSSION

This study explores the multifaceted challenges African women in surgery encounter. A central theme emerging from this study is the delicate balance women in surgery must strike between their professional and personal lives. Heavy workloads and long working hours were reported as significant barriers underscoring the demanding nature of surgical careers. These demands can create conflicts with cultural and societal expectations prevalent in African societies, where women traditionally hold roles as primary caretakers and homemakers.^11^ Additionally, the confusion between female surgeons and nurses, despite their professional appearance, complicates their roles and recognition in the hospital setting. Such gender-related stereotypes and consequences, such as lowered morale or hampered self-confidence, have similarly been described in contexts beyond the African region.^19^

Pursuing a career in surgery can be perceived as neglecting these roles, leading to societal disapproval. The presence of a “marriage wage penalty” and a “motherhood wage penalty” indicates the systemic challenges faced by women in surgery, especially when they enter marriage or have children.^12^ The narrative that women must prioritise family life over their surgical careers is deeply integrated into cultural and societal expectations. Such attitudes place an additional burden on women, potentially leading to their exit from surgical training. These findings confirm reports from previous studies in Rwanda^13^, South Africa ^14^, and Australia.^15^

The financial aspect of these barriers and obstacles is a critical element that deserves attention. Although this study did not assess the cost of training, respondents identified this as a barrier. O’Callaghan et al. surveyed medical students and surgeons in the UK and found a substantial cost associated with training.^16^ Little is known about the cost of surgical training in Africa. However, in a society where more educational and training opportunities are given to boys, it is plausible that the cost associated with surgical training can be a severe limiting factor for women with surgical career aspirations.

This study uncovers the pervasive gender bias and discrimination that women in surgical training endure. The mistreatment they face is broad and includes underestimation of their skills, unequal opportunities in the workplace, mansplaining, unsolicited advice, microaggressions, sexual harassment, and sexist jokes. These findings echo previous research in Nigeria and South Africa, where the women in surgery reported having experienced some form of gender bias and discrimination.^14,17^ Laessig et al.^18^ also emphasised the presence of gender bias during training. One example described was that letters of recommendation for female surgeons often focused on their physical appearance rather than their clinical competence. This underscores the importance of addressing gender bias at various stages of surgical careers, from training to career advancement.

Some respondents reported religion to have been one of the barriers that prevented women from pursuing surgical careers. Although we could not elaborate further in this study, this finding resonates with previous studies documenting religion as a potential obstacle to female advancement into surgical professions.^4,20,21^

Despite the numerous barriers women face in surgery in Africa, this study identifies potential solutions and strategies to mitigate these. These include encouragement and support from the community and family to fulfil their aspirations while maintaining a healthy work-life balance. These efforts should also encompass mentorship, leadership roles for women in surgery, and confidence building. Providing financial support for female surgeons in training can also help alleviate the financial constraints associated with surgical careers. A fundamental shift in attitude within the surgical community is also essential. Colleagues, peers, and employers should actively support and hold accountable those who engage in discriminatory practices, harassment, and bias. Efforts should be made to allow flexible work arrangements and structured residency programs to help women pursue surgical careers without sacrificing their personal lives. Finally, the academic, healthcare, and surgical communities should work together to develop data-driven solutions and policy changes that promote gender equity.

### Strengths and limitations

This study’s strength lies in its diverse participant pool, from various surgical backgrounds and cadres across Africa. It provides a comprehensive mapping of a broad range of challenges and opportunities and lays the groundwork for future in-depth interview studies. However, the primary limitations include a small sample size and a majority of medical student participants, limiting insights into some critical barriers. Additionally, responder bias may influence results, as those with interest or experience in gender discrimination may be more or less likely to participate.

## CONCLUSION

This study offers a comprehensive understanding of the barriers faced by women in surgery in Africa, encompassing issues of work-life balance, hospital culture, financial barriers, and societal and religious expectations. It highlights the need for concerted efforts to overcome these challenges and foster an empowering environment that allows women to pursue their surgical careers with due recognition, respect, and support.

## References

[1] Bellini MI, Graham Y, Hayes C, Zakeri R, Parks R, Papalois V. A woman’s place is in theatre: women’s perceptions and experiences of working in surgery from the Association of Surgeons of Great Britain and Ireland women in surgery working group. BMJ Open. 2019 Jan;9(1):e024349. 10.1136/bmjopen-2018-024349 PMC6326292 30617103

[2] Mahoney ST, Strassle PD, Schroen AT, Agans RP, Turner PL, Meyer AA, et al. Survey of the US Surgeon Workforce: Practice Characteristics, Job Satisfaction, and Reasons for Leaving Surgery. J Am Coll Surg. 2020 Mar;230(3):283–293.e1. 31931143 10.1016/j.jamcollsurg.2019.12.003

[3] Malik M, Inam H, Janjua MBN, Martins RS, Zahid N, Khan S, et al. Factors Affecting Women Surgeons’ Careers in Low–Middle-Income Countries: An International Survey. World j surg. 2021 Feb;45(2):362–8. 33040193 10.1007/s00268-020-05811-9

[4] O’Flynn E, Andrew J, Hutch A, Kelly C, Jani P, Kakande I, et al. The Specialist Surgeon Workforce in East, Central and Southern Africa: A Situation Analysis. World j surg. 2016 Nov;40(11):2620–7. 27283189 10.1007/s00268-016-3601-3

[5] Ozgediz D, Riviello R, Rogers SO. The surgical workforce crisis in Africa: a call to action. Bull Am Coll Surg. 2008 Aug;93(8):10–6. 19492736

[6] Odera A, Tierney S, Mangaoang D, Mugwe R, Sanfey H. Women in Surgery Africa and research. The Lancet. 2019 May;393(10186):2120. 10.1016/S0140-6736(19)31106-7 31226043

[7] Jesuyajolu DA, Okeke CA, Obuh O. The Challenges Experienced By Female Surgeons in Africa: A Systematic Review. World j surg. 2022 Oct;46(10):2310–6. 35789283 10.1007/s00268-022-06650-6

[8] ALobaid AM, Gosling C, Khasawneh E, McKenna L, Williams B. Challenges Faced by Female Healthcare Professionals in the Workforce: A Scoping Review. JMDH. 2020 Aug; Volume 13:681–91. 32821112 10.2147/JMDH.S254922 PMC7417925

[9] Braun V, Clarke V. Thematic analysis. In: Cooper H, Camic PM, Long DL, Panter AT, Rindskopf D, Sher KJ, editors. APA handbook of research methods in psychology, Vol 2: Research designs: Quantitative, qualitative, neuropsychological, and biological [Internet]. Washington: American Psychological Association; 2012 [cited 2024 Jan 4]. p. 57–71. Available from: http://content.apa.org/books/13620-004

[10] Kobayashi AK. Women in thoracic surgery: Asian perspective. J Thorac Dis. 2021 Jan;13(1):456–9. 33569231 10.21037/jtd-2020-wts-04 PMC7867803

[11] African Development Bank and United Nations Economic Commission for Africa. Africa Gender Index Report 2019; What the 2019 Africa Gender Index tells us about gender equality, and how can it be achieved [Internet]. Africa Development Bank; 2020. [cited 2023 Dec 20] Available from: https://www.afdb.org/sites/default/files/documents/publications/africa_gender_index_report_2019_-_analytical_report.pdf

[12] Glauber R. Marriage and the Motherhood Wage Penalty Among African Americans, Hispanics, and Whites. J of Marriage and Family. 2007 Nov;69(4):951–61.

[13] Yi S, Lin Y, Kansayisa G, Costas-Chavarri A. A qualitative study on perceptions of surgical careers in Rwanda: A gender-based approach. Koniaris LG, editor. PLoS ONE. 2018 May 10;13(5):e0197290. 10.1371/journal.pone.0197290 PMC5944995 29746556

[14] Naidu P, Mazza IB. Surgery in South Africa - challenges and barriers. S Afr j surg [Internet]. 2021 [cited 2024 Jan 4];59(3). Available from: http://ref.scielo.org/zn3g4t 34515420

[15] Liang R, Dornan T, Nestel D. Why do women leave surgical training? A qualitative and feminist study. The Lancet. 2019 Feb;393(10171):541–9. 10.1016/S0140-6736(18)32612-6 30739689

[16] O’Callaghan J, Mohan HM, Sharrock A, Gokani V, Fitzgerald JE, Williams AP, et al. Cross-sectional study of the financial cost of training to the surgical trainee in the UK and Ireland. BMJ Open. 2017 Nov;7(11):e018086. 10.1136/bmjopen-2017-018086 PMC5695344 29146646

[17] Adebola SN, Jamila L, Rasheedat S, Abdulrahman AO, Bello FM. Women in Otorhinolaryngolgy in Nigeria. Int J Otorhinolaryngol Head Neck Surg. 2021 Feb 24;7(3):426.

[18] Laessig M, Ullrich L, J. Papadimos T, A. Handspiker E, A. Cama C, P. Stawicki S. Surgical Education: Focus on Gender Equality in Academic Surgery and Related Areas. In: P. Stawicki S, S. Firstenberg M, P. Orlando J, J. Papadimos T, editors. Contemporary Topics in Graduate Medical Education - Volume 2 [Internet]. IntechOpen; 2022 [cited 2024 Jan 4]. Available from: https://www.intechopen.com/chapters/81943

[19] Teresa-Morales C, Rodríguez-Pérez M, Araujo-Hernández M, Feria-Ramírez C. Current Stereotypes Associated with Nursing and Nursing Professionals: An Integrative Review. IJERPH. 2022 Jun 22;19(13):7640. 35805296 10.3390/ijerph19137640 PMC9265497

[20] Sarmiento Altamirano D, Himmler A, Cabrera Ordoñez C, Olmedo Abril S, Biondi A, Di Saverio S. Gender disparities in Ecuador: a survey study of the under-representation of women in surgery. Updates Surg. 2021 Oct;73(5):2009–15. 33464475 10.1007/s13304-020-00964-7

[21] Makama JG, Garba ES, Ameh EA. Under Representation of Women in Surgery in Nigeria: By Choice or by Design?. Oman Med J. 2012 Jan 16;27(1):66–9 22359731 10.5001/omj.2012.15 PMC3282129

